# Effect of Asphaltenes on the Stability of Water in Crude Oil Emulsions

**DOI:** 10.3390/ma18030630

**Published:** 2025-01-30

**Authors:** Yan Peng, Xiangyu Zhang, Lihua Cheng, Hong Zhang, Jieyun Tang, Hong Chen, Qinzhen Fan, Xinping Ouyang

**Affiliations:** 1 School of Chemical Engineering, Guangdong University of Petrochemical Technology, Maoming 525000, China; pengyan.lpec@sinopec.com (Y.P.); chenlh@gdupt.edu.cn (L.C.); chenhonggds@163.com (H.C.); 2 School of Chemistry & Chemical Engineering, South China University of Technology, Guangzhou 510640, China; 3 Research Institute of China National Petroleum Corp Liaohe Oilfield, Panjin 124010, China; zhangzy1@petrochina.com.cn (X.Z.); zhangh001@petrochina.com.cn (H.Z.);

**Keywords:** heavy crude oil, asphaltenes, emulsion, aggregates, critical aggregation concentration

## Abstract

The presence of asphaltene, especially in heavy crude oil, causes difficulties in the de-watering/desalting process, which is the initial step of crude oil pretreatment. This study investigates the effect of asphaltenes on the stability of crude oil emulsions using a simulated oil system composed of toluene and n-heptane. It was found that asphaltenes behave like conventional surfactants, adsorbing at the oil–water interface and reducing interfacial tension. The critical aggregation concentration (CAC) of W/O emulsions formed from a toluene and n-heptane mixture (7:3 volume ratio) was found to be 0.05 g/L. When the asphaltene concentration was greater than CAC, the asphaltene aggregated into clusters, forming a viscoelastic interface film that enhanced the strength of the emulsion droplets. At an asphaltene concentration of 0.01 g/L, the storage modulus (G′) and loss modulus (G″) were 1.12 Pa·s and 8.94 Pa·s, respectively. The storage modulus G′ was less than the loss modulus G″, indicating that the viscoelastic nature of the emulsion, and both the G′ and G″ of the emulsions increased with the increasing asphaltene concentration. When the concentration reached 11 g/L, G′ and G″ were 1033 Pa·s and 416 Pa·s, respectively, with G′ exceeding G″, indicating that the emulsion became more stable. Moreover, increasing the solvent aromaticity led to a rise in the CAC of asphaltene, which was favorable for breaking emulsions. The findings imply that reducing the asphaltene content at the interfaces of oil–water by adding an aromatic solvent or blending with light crude oil is a feasible way to break the emulsion and further dewater and desalt.

## 1. Introduction

With rising global demand for crude oil, there is a growing emphasis on the development of heavy crude oil as a prospective future contributor to incremental oil and gas production [1]. Among the components of heavy crude oil, asphaltene stands out as the most polar and heavy fraction [2]. Asphaltenes are high-molecular-weight components composed of nonpolar polyaromatic rings and alkane chains of varying lengths, along with organic polar fractions containing sulfur, nitrogen, and oxygen functional groups. The amphiphilic structure of asphaltene molecules enabled them to behave like surfactants, allowing interaction with both the nonpolar oil phase and the polar water phase. [3]. Moreover, the elongated side chains present in the asphaltene molecular structure provide a certain viscoelasticity. Therefore, when water is added to crude oil, asphaltene acts as a natural surfactant, forming a stable interfacial membrane that enhances the emulsion’s stability [4,5,6,7]. Unfortunately, the electric desalting and dewatering processes employed during crude oil refining serve as preliminary steps to avoid corroding the process equipment and poisoning catalysts in downstream refineries [1]. In the desalting process, water is deliberately mixed with crude oil to dissolve hydrophilic substances and generate water-in-oil (W/O) emulsions. The emulsions had to subsequently be broken down to remove both salt and water [8,9]. However, the presence of asphaltene significantly hinders the desalting and dewatering processes, as it enhances emulsion stability.

In recent years, tremendous efforts have been devoted to investigating the stabilization mechanism of asphaltenes on oil/water emulsions for regulating phase separation in these systems. Liu et al. [10] investigated effective strategies to mitigate reservoir choke caused by emulsification or the Jamin effect using light crude oil dilution. Their results indicated that the size distribution range of W/O droplets increased with higher water fractions, and larger W/O droplets tended to break first during dilution. Several studies have emphasized that the organic polar fractions of crude oil, particularly asphaltenes and resins, play a significant role in its stability. Furthermore, mobility control mechanisms can effectively enhance oil recovery when emulsions are generated during enhanced oil recovery (EOR) operations [11,12,13].

Morimoto et al. [14] investigated the effects of aromaticity, solubility parameters, and dosages of stimulated oils on the self-assemblies of asphaltenes. The Yen–Mullins model [15] revealed the influence of n-heptane addition on the stability of W/O emulsions and the kinetics of asphaltene aggregation in organic solvents. The addition of n-heptane led to the depolymerization of the aggregates of asphaltenes. With increasing the concentration of asphaltenes, the process of aggregate formation shifted from a diffusion-limited aggregation into a reaction-limited aggregation mechanism. Alhreez et al. [16] studied the detailed molecular structure of asphaltenes in oil/water emulsions and proposed a mechanism for their structural modification and the stabilization of asphaltenes. Adding asphaltene inhibitors increased the stacking distance between aromatic rings and aliphatic chains in asphaltene, which reduced the aromatic sheet diameter and the cluster size. These structural alterations led to a decrease in asphaltene aromaticity, consequently increasing the solubility of aromatic compounds in the oil. Furthermore, the disruption of the aromaticity prevented π-π stacking between asphaltene molecules [17], which was responsible for reducing emulsion stability and contributed to the dewatering of the heavy crude oil.

Andreatta et al. [18] suggested that asphaltenes exhibited surfactant-like behavior and formed micelle-like aggregates at concentrations above a certain threshold. The critical aggregation concentration (CAC) of asphaltenes in toluene was about 0.10 g/L. The asphaltenes, which were in the form of colloidal nano-aggregates, had a strong capability to persist at the oil/water interface, forming a rigid interfacial film [19,20]. However, it was not clear regarding the mechanism of action of asphaltene in crude oil emulsion due to the complicated structure of asphaltene [21]. This work aimed to investigate the aggregation phenomenon of asphaltenes in oil/water emulsions using a mixture of toluene and n-heptane as a model for crude oil. Additionally, the impact of aromaticity on the stability of W/O emulsions containing asphaltenes by adjusting the ratio of toluene to n-heptane was explored to provide valuable insights for the desalting and dewatering of heavy oil.

## 2. Materials and Methods

### 2.1. Materials

Toluene (99.8%) was purchased from Guangdong Guangxing Science and Technology Co. Ltd., Guangzhou, China. N-heptane (98%) was supplied by Aladdin Chemical Reagent Co. Ltd., Beijing, China. The crude oil used in this work was produced from Napo oriente, Tena, Ecuador, and its composition is listed in Table 1, which indicates that it was a typical heavy oil with high asphaltene content.

Asphaltenes were isolated from the Napo oriente crude oil using n-heptane as a precipitant. In brief, for the Napo oriente crude oil, the asphaltenes were prepared following the ASTM D 7996 method [27]. One g of crude oil was mixed with 40 mL of n-heptane, and the mixture was stirred at 300 rpm for 1 h at room temperature (25 °C) to form a homogeneous solution. The solution was then allowed to equilibrate for 24 h in a dark environment. Subsequently, the mixture was filtered through filter paper to separate the precipitated asphaltene particles. The asphaltene solid was washed five times with 100 mL of n-heptane to remove any soluble components. Finally, the washed asphaltenes were dried under vacuum at 60 °C for 12 h [28]. The obtained asphaltene powder is a mixture of high-molecular compounds characterized by polycyclic aromatic hydrocarbon structures, along with functional groups such as nitrogen, oxygen, and sulfur. The composition analysis of the asphaltenes was performed using an elemental analyzer (Vario ELcube, Elementar Co., Frankfurt, Germany), and the results are shown in Table 2.

### 2.2. Emulsion Preparation and Characterization

The W/O emulsions were prepared via a high-shear stirring emulsification method. The oil phase was formed by completely dissolving asphaltenes in toluene/n-heptane solvents. The prepared asphaltene solid powder was used to prepare W/O emulsions with asphaltene concentrations ranging from 0.01 to 11.00 g/L, consisting of a 7:3 volume ratio of toluene to n-heptane using an Ultra-Turrax emulsifier (IKA-based, IKA Co., Staufen, Germany) at 8000 rpm for 10 min at room temperature (25 °C). The stability of the emulsion was determined based on dewatering percent (X), which was calculated as follows:

(1) $$X=V/V_{0}\times 100\%$$

where X is the dewatering percentage and $V_{0}$ and V are the water volumes of the initial addition and the free water separated, respectively.

A polarized light microscope with a digital camera (Axioskop40POL, Zeiss Co., Oberkochen, Germany) was used to examine the distribution and shape of an emulsion droplet. The stability of all emulsions was evaluated with the Turbiscan LabExpert stability analyzer (Expert model, Formulaction Co., Toulouse, France). The morphology of the asphaltenes in a W/O emulsion was characterized by scanning electron microscopy (Merlin, Carl Zeiss, Oberkochen, Germany).

### 2.3. Rheological Performance Test

The shear viscosity and oscillatory scan tests of the emulsions were measured by a rheometer (MARS III, Haake Co., Karlsruhe, Germany) using a conical plate rotor with a cone angle of 1° at room temperature (25 °C). The gap was set to 0.052 mm. The shear viscosity flow tests were performed by increasing the shear rate from 1 s^−1^ to 10 s^−1^, and the apparent viscosity values were recorded. As for the oscillation strain sweep experiments, the samples were first subjected to a stress sweep test in the oscillation strain ranging from 0.01 to 100%, and the frequency was 1 Hz to determine the linear viscoelastic region (LVR) of the emulsion. The conditions of the oscillation amplitude test were as follows: angular frequency 0.1 rad/s to 100 rad/s and strain 1%. The storage modulus (G′) and loss modulus (G″) were calculated from the strain response.

### 2.4. Fluorescence Spectrometry

The samples were excited using a steady-state fluorescence spectrometer (F-4600, Hitachi Co., Tokyo, Japan). The scan range was set from 350 to 700 nm with a scan speed set to 1200 nm/min at room temperature (25 °C). The slit width and excitation wavelength were set to 5 nm and 352 nm, respectively.

### 2.5. Characterization of the Water/Oil Interface

The interfacial tension between the model oil containing asphaltene and a combination of toluene and n-heptane, as well as pure water, was measured using a fully automated surface tension meter (BZY-1 type, Shanghai Hengping Instrument Factory, Shanghai, China) with the Wilhelmy plate method. The experiment was repeated at least three times to obtain the results, including the mean and standard deviation.

The interfacial expansion modulus of the oil/water interface was carried out using an Interfacial Swelling Rheometer (JMP2000, Shanghai Zhongchen Digital Technology Equipment Co., Ltd., Shanghai, China) by imposing periodic compression and expansion within a frequency range of 0.01–0.20 Hz and a small sinusoidal deformation of 10 % at room temperature (25 °C). The corresponding changes in interfacial tension were observed, and the interfacial swelling elasticity modulus was recorded.

## 3. Results

### 3.1. Effect of Asphaltene Content on the Stability of W/O Emulsions

**Figure 1:**
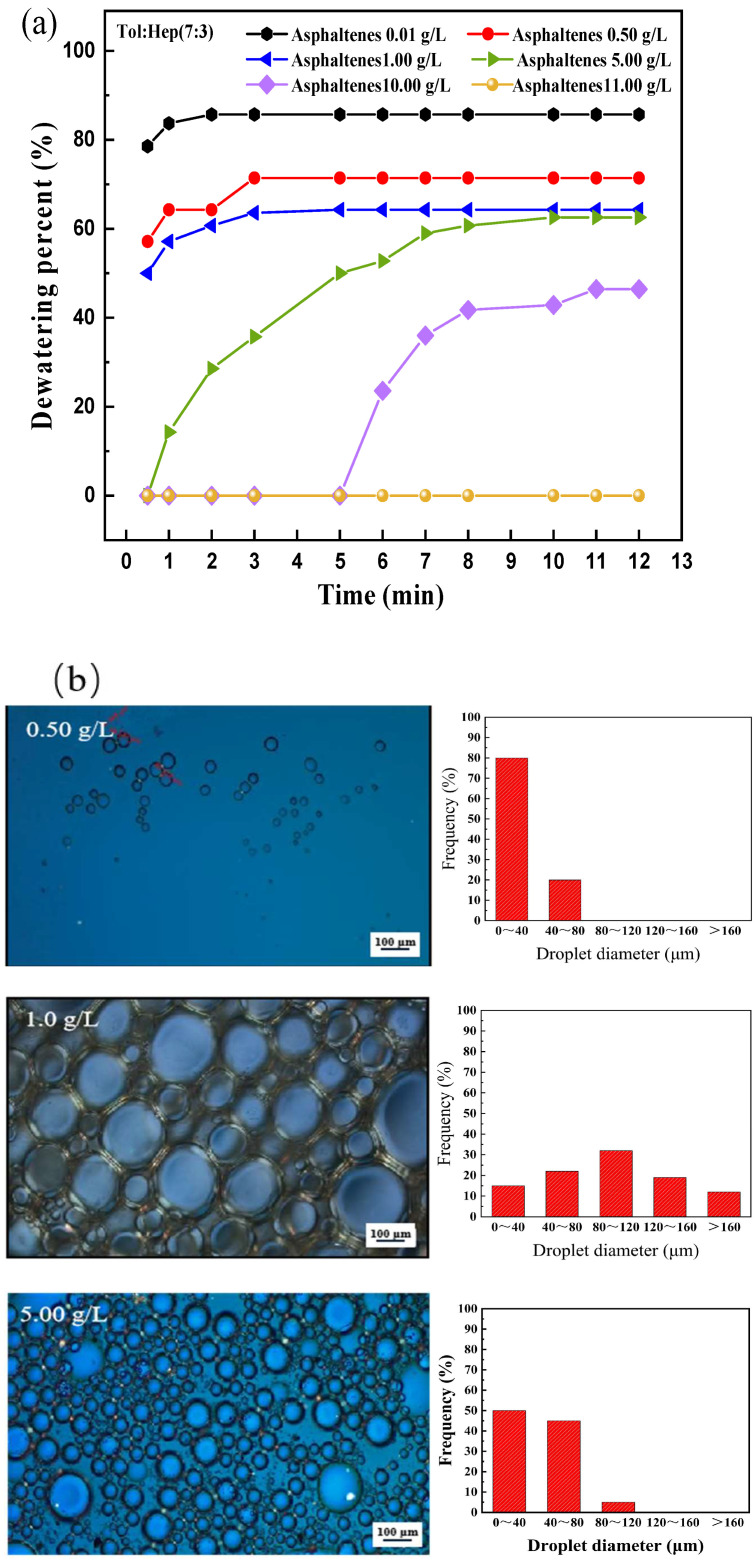

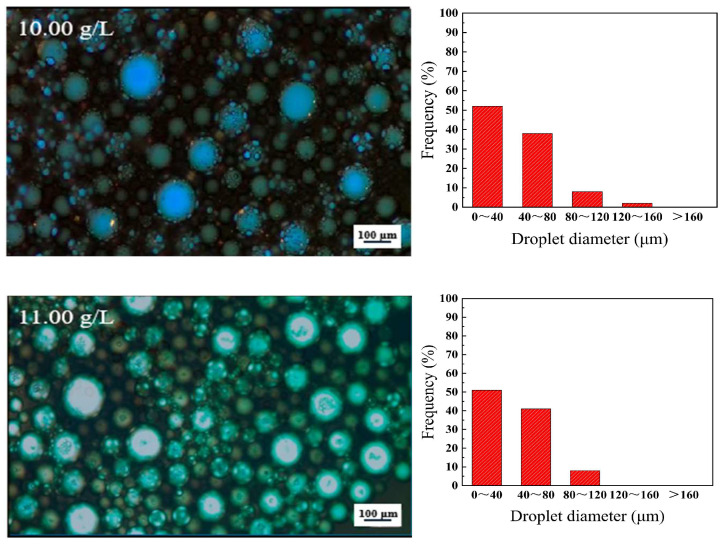
Effect of asphaltene contents on the stability (**a**) and microstructures (**b**) of W/O emulsion. (W/O emulsion included 20 mL with different asphaltene contents ranging from 0.01 to 11.00 g/L consisting of a 7:3 volume ratio of toluene to n-heptane and 6 mL of water. The microscope images were taken after emulsions had been formed for 15 min).

The effect of the asphaltene contents on the stability and micromorphology of the emulsions composed of 20 mL of model oil, and 6 mL of water are shown in Figure 1.

Several factors were influenced by the emulsification of water in crude oil, including the asphaltenes, resins, waxes, and mineral fines present in crude oil. As natural surface-active agents, asphaltenes were significantly involved in the interfacial tension between the water and oil phases and played a crucial role in the emulsification process [10,11]. It is found from Figure 1a that, with the increase in asphaltene content, the dewatering percent decreased and the time to reach dewatering equilibrium was prolonged, indicating that asphaltene was effective in preventing water removal from the emulsion system, hence improving the stability of the emulsion [29]. When the content of asphaltene was 0.50 g/L, the dewatering percent was 67% after 3 min of standing. By further increasing the asphaltene concentration to 11.00 g/L, the dewatering percent reduced to zero, indicating that the emulsion reached a relatively stable state.

The microscope images presented in Figure 1b reveal that, when the asphaltene concentration was 0.50 g/L, there were a small number of emulsion droplets existing in the W/O system. When the concentration of asphaltene increased to 1.00 g/L, a lot of the larger emulsion droplets were observed between the model oil and water. With a further increase in the dosage of asphaltene to over 5.00 g/L, the emulsion droplets became smaller and more uniform, implying an enhanced stability of the emulsion. Additionally, when the concentration of asphaltene was greater than 1.00 g/L, a distinct interfacial film layer could be seen at the boundary of the emulsion droplets, whose thickness increased with the rising asphaltene concentration. This meant that asphaltene served as the natural emulsifier and bolstered the stability of the emulsion system, resulting in difficulties in dewatering from the W/O emulsion system.

**Figure 2:**
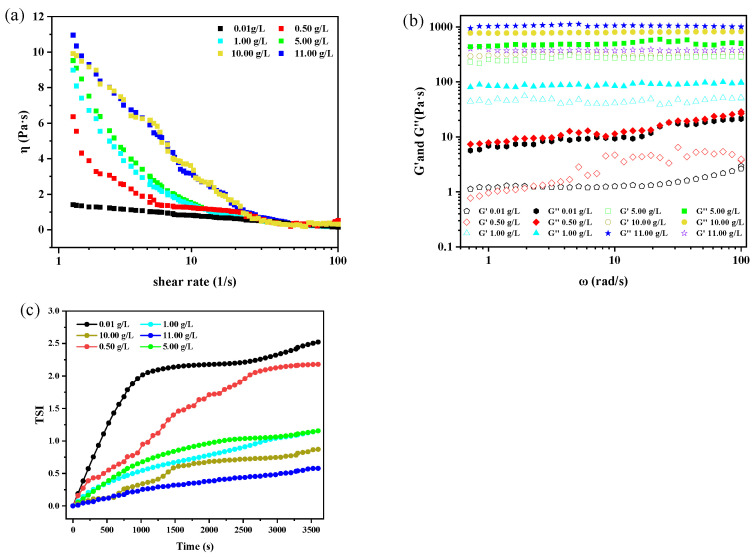
Rheological properties and TSI values of W/O emulsions with the presence of different content asphaltenes. (**a**) Shear viscosity, (**b**) energy storage modulus G′ and loss modulus G″, and (**c**) TSI values. (W/O emulsions were composed of the mixtures of toluene and n-heptane at 7:3 volume ratio, asphaltene, and 6 mL water).

To explore the mechanism of the stability of emulsion improved by asphaltene, the rheological properties and TSI values [30] of W/O emulsions were tested and shown in Figure 2.

It is found from Figure 2a that the viscosity of the emulsion increased with an increasing asphaltene concentration and decreased with an increasing shear rate, exhibiting a typical shear-thinning non-Newtonian fluid behavior [31,32].

Jansen et al. [33] proposed a scaling parameter *Fl_d_* for evaluating the viscosity of the dispersion system.

(2) $$Fl_{d}=\frac{4\pi \eta_{s}\gamma \alpha^{2}\alpha_{m}}{kT\varphi_{m}}$$

where $\eta_{s}$, γ, *k*, α, $\alpha_{m}$, and $\varphi_{m}$ were the solvent viscosity, shear stress, thermodynamic constant, dispersed phase droplet radius, micelle radius, and micelle volume fiction, respectively.

Based on the Equation (2), the increase in asphaltene concentration resulted in the decrease of γ, $\alpha^{2}$, and $\alpha_{m}$ and an increase of $\varphi_{m}$ (Figure 1b) and, hence, a decreased $Fl_{d}$ value. Consequently, the viscosity of the W/O emulsions decreases with the increase of asphaltene concentration.

The viscosity of emulsions with different dosages of asphaltene decreased with an increasing shear rate, which was attributable to the increased degree of orientation along the shear direction in the asphaltene molecules, which reduced the long-chain structure of the asphaltene intermolecular associations and the liquid layer entanglement, thereby reducing flow resistance [34]. Moreover, the decrease in viscosity could be caused by the disruption of asphaltene agglomerates under shear stress. Figure 2b presented the double-logarithmic plots of storage moduli (G′) and loss moduli (G″) of the emulsions with different contents of asphaltene as a function of frequency sweeping from 0.1 to 100 rad/s at a constant strain of 1%, in which every G′ was less than G″, indicating a viscoelastic behavior of the emulsion. Additionally, both G′ and G″ increased with an increasing asphaltene concentration, and when the asphaltene dosage reached or exceeded 1.00 g/L, the G′ and G″ remained nearly constant with the changes in frequency, suggesting that the emulsions were stable and unaffected by the shear forces due to the formation of interfacial films with a certain strength, which could prevent droplet coalescence effectively [35,36]. Figure 2c shows that the TSI values of the emulsions decreased with a rising asphaltene concentration, indicating that the stability of the emulsion was enhanced by the increased asphaltene dosage because a low TSI value meant that a suspension had good stability. The three-dimensional network structure of asphaltene provided the emulsion interfacial film greater rigidity, improved emulsion viscosity, slowed down the migration speed and collision of oil droplets, and ultimately enhanced the stability of the emulsion. These results were in agreement with that of the dewatering of the emulsion (Figure 1a).

### 3.2. Effect of Aromaticity of Simulated Oil on the Stability of Asphaltene-Containing Emulsions

**Figure 3:**
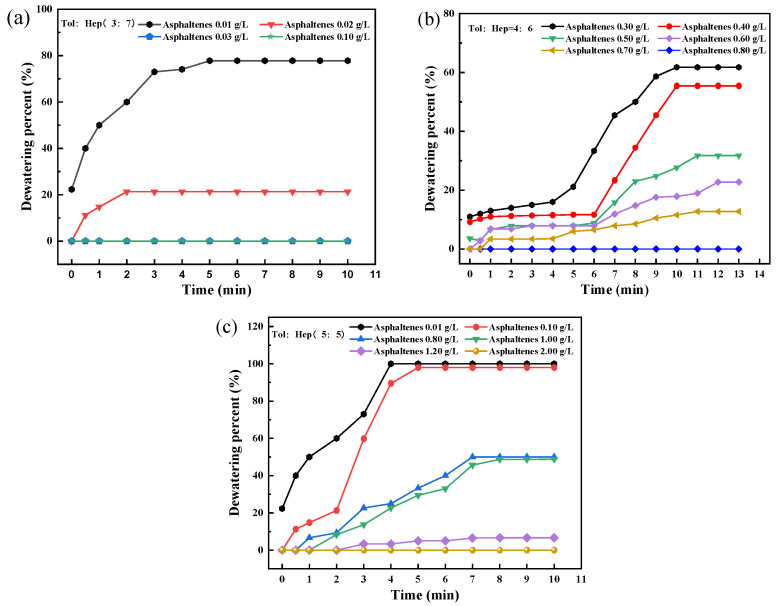
Dewatering percent of the W/O emulsion with different aromaticity of simulated oil (**a**) tol:hep (3:7), (**b**) tol:hep (4:6), and (**c**) tol:hep (5:5).

The aromaticity of simulated oil was regulated by changing the ratio of toluene to n-heptane. The effect of the aromaticity of the simulated oil on the stability of asphaltene-containing emulsions, as judged by dewatering percent, is shown in Figure 3.

As shown in Figure 3, it was found that, in the case of the volume ratio of toluene to n-heptane in the oil being 3:7, a fully emulsified W/O emulsion without dewatering was achieved at the asphaltene dosage exceeding 0.03 g/L. However, when the volume ratios of toluene to n-heptane in the oil increased to 4:6 and 5:5, the lowest asphaltene dosages were 0.80 g/L and 2.00 g/L, respectively. These results indicated that the emulsifying ability of asphaltene significantly decreased with increasing aromaticity of the oil phase.

**Figure 4:**
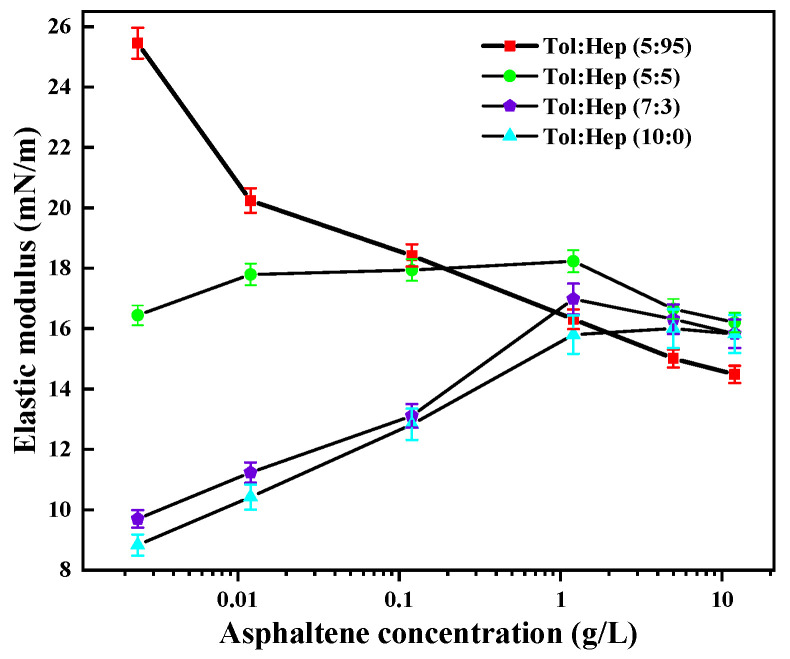
Elastic modulus of mixtures containing asphaltenes and simulated oil with different aromaticity.

The elastic modulus of the mixtures of asphaltene and simulated oil with varying aromaticities was determined, and the results are shown in Figure 4.

At the asphaltene concentration below 0.11 g/L, the elastic modulus decreased gradually with the increasing aromaticity of the simulated oil. It is generally believed that asphaltenes were mainly composed of aromatic rings, and an increase of aromaticity in the simulated oil possibly resulted in the partial dissolution of asphaltenes to a certain degree, leading to a decrease in the elastic modulus and stability of the emulsion. Under high asphaltene content above 10.00 g/L, simulated oils with different aromaticities exhibited a similar modulus except for low-aromaticity oil. This revealed that the aromaticity of the oils has less impact on the stability of the emulsion with a high concentration of asphaltene. However, it was worth noting that low-aromaticity oil with toluene contents of less than 5% exhibited a very low elastic modulus even at higher asphaltene contents (10.00 g/L). This was due to the deposition of asphaltene resulting from a high asphaltene concentration and a low content of aromatic hydrocarbons in the oil phase, which further reduced the emulsion stability. These imply that the dewatering of crude oils with high asphaltene content can be manipulated by adding aromatics or blending crude oils with a high arene content.

### 3.3. Mechanism of Asphaltene at the Oil/Water Interface

**Figure 5:**
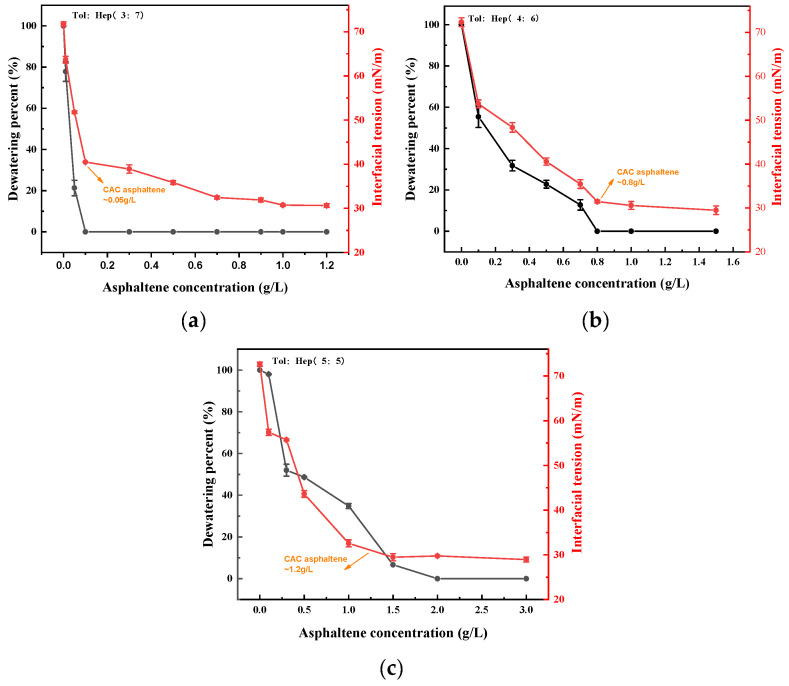
Effect of asphaltene concentration on oil–water interfacial tensions and dewatering percent after 15 min standing of emulsion: (**a**) tol:hep (3:7), (**b**) tol:hep (4:6), and (**c**) tol:hep (5:5).

The effects of asphaltene concentration on the interface tension of a W/O emulsion are shown in Figure 5, in which the ratio of toluene to n-heptane ranged from 3:7 to 5:5.

Figure 5 illustrated that the interfacial tensions of the simulated oil–water emulsions decreased rapidly with an increasing asphaltene concentration, and then reached a plateau value. These trends were similar to those of conventional surfactants [37,38]. Asphaltene exhibited amphiphilic characteristics, where the lipophilic nonpolar sites of asphaltene molecules were attracted to the oil phase. Meanwhile, the hydrophilic polar sites were bound to the water phase in the emulsion, forming an oriental absorption film that decreases the interfacial tension. With a further increase in the asphaltene concentration, asphaltene began to aggregate. At this stage, the interfacial tension no longer decreased, and the critical aggregation concentration (CAC) was observed as the asphaltene transitioned into an aggregated state with a further increase in concentration. Consequently, the thickness of the interfacial film increased (Figure 1), and hence, the stability of the emulsion increased. It was also found from Figure 5 that there was a consistent effect of asphaltene concentration on both the interfacial tension and dewatering percentage of the emulsion. When the asphaltene concentration reached the CAC, both the interfacial tension and dewatering percent remained almost unchanged. Asphaltene accumulates at the W/O interface, forming a viscoelastic interface film that stabilizes the emulsions and reduces droplet coalescence [28]. Figure 5 also demonstrates that the CAC value of asphaltenes was higher when the solvent aromaticity was higher (higher content of toluene). It was recognized that asphaltenes were easily dissolved in the aromatic solvents, and the dissolved asphaltenes entered into the bulk oil phase from the interfaces, resulting in a decrease in the stability of the emulsion [39]. This finding suggested that the dewatering process of crude oil can be improved by increasing the aromatic solvent or mixing it with crude oil with high aromaticity.

**Figure 6:**
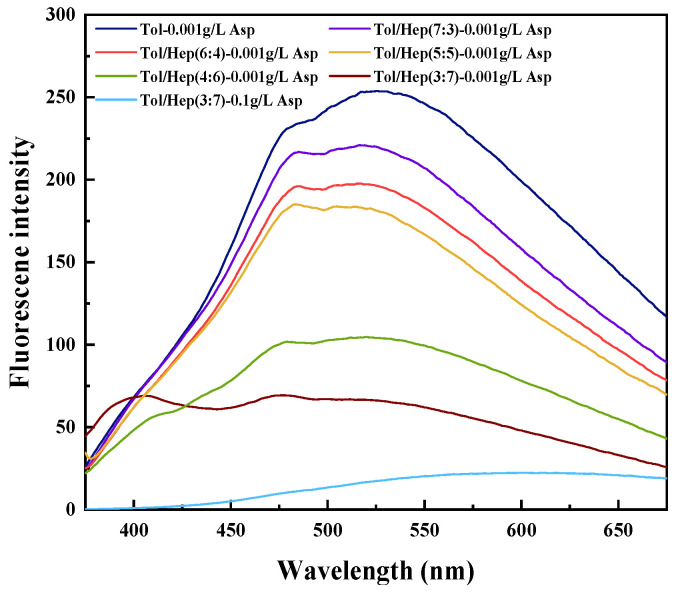
Fluorescence spectra of asphaltene in simulated oil with different solvent aromaticity excited at λ = 352 nm.

Fluorescence spectroscopy was used to investigate the effect of the aromaticity of the simulated oil on the aggregation behavior of asphaltene molecules, and the results are shown in Figure 6.

It was generally considered that the aggregation mechanism of asphaltene was associated with molecular hydrogen bonding and π-π stacking among aromatic rings, and the greater the fluorescence quenching, the stronger the aggregation between molecules [40,41]. When the concentration of asphaltene was 0.001 g/L, the fluorescence intensity gradually weakened and blue-shifted with the decrease of aromaticity, suggesting an increase in self-aggregation among asphaltene molecules in the simulated oil [42,43]. In a mixture comprising toluene and n-heptane at a ratio of 3:7, when the concentration of asphaltenes increased to 0.10 g/L, the asphaltene aggregations were further intensified, and therefore, fluorescence quenching occurred [44].

**Figure 7:**
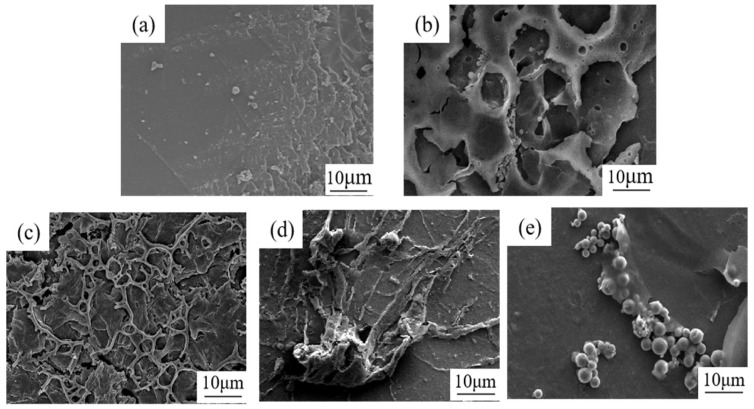
SEM images of the interfacial membrane at different contents of toluene in a simulated oil–water system: (**a**) tol:hep (10:0), (**b**) tol:hep (7:3), (**c**) tol:hep (5:5), (**d**) tol:hep (4:6), and (**e**) tol:hep (3:7).

To analyze the interfacial film, different volumes of n-heptane solvent were added dropwise to a 0.05 g/L toluene solution containing asphaltene at the same stirring speed and mixed thoroughly with water. One mL of the simulated oil–water mixture at the oil–water interface was sucked out for SEM imaging, and the result is shown in Figure 7.

When the oil phase consisted of pure toluene, asphaltenes were well dispersed within the oil phase, and no asphaltene aggregates were observed at the interface. (Figure 7a). When the volume fraction of toluene was 70%, there was still a significant amount of toluene, which resulted in the partial asphaltene dissolution and the absorption of some asphaltene aggregated at the oil–water interface (Figure 7b).

However, at 50 vol% toluene, a significant amount of stacked three-dimensional skeleton structures formed due to the entanglement of the branched chains of asphaltene (Figure 7c). When the toluene content was lower than 50 vol%, more asphaltenes were transferred from the oil phase to the interface (Figure 7d), resulting in the formation of larger asphaltene aggregates in the interfacial film. With the decrease in aromaticity of stimulated oil, a lot of asphaltenes were absorbed at the interface, forming globules that aggregated to form an interfacial film (Figure 7e).

**Figure 8:**
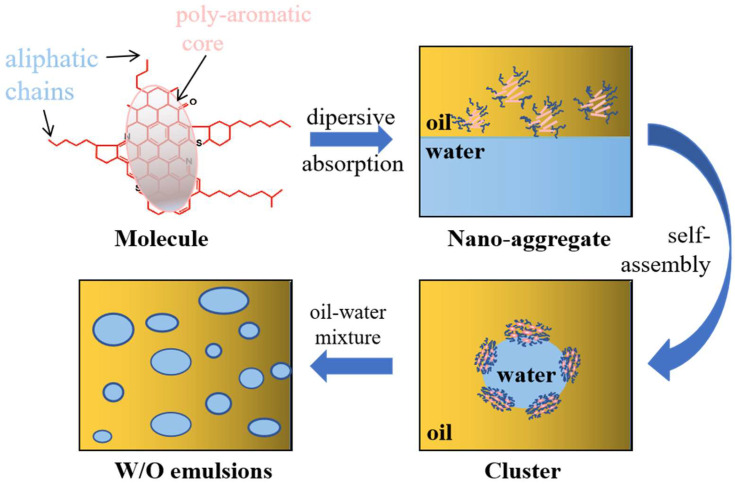
Mechanism of 0.001 g/L asphaltene in stabilization of simulated oil–water emulsions.

Based on the Yen model [45], a mechanism of emulsion stability enhanced by asphaltene was proposed, as shown in Figure 8.

In the emulsion system, asphaltenes were dispersed in the oil phase and adsorbed at the oil–water interface. The interactions between asphaltene molecules, such as π-π stacking, hydrogen bonding, and dipole interactions, led to the formation of nano-aggregates. When the concentration of asphaltenes was sufficiently high, these nano-aggregates self-assembled into clusters, further forming a viscoelastic interface film that stabilized the emulsion under certain mixing conditions [6,46]. Therefore, the asphaltene content played a crucial role in the stability of water-in-oil (W/O) emulsions. The presence of asphaltenes significantly affected the dewatering and desalting processes of crude oil, as stable emulsions complicated the removal of water and salts. Consequently, breaking the emulsion facilitated the dewatering and desalting of heavy crude oil, which could be achieved by adding aromatic solvents or blending with low-asphaltene crude oil.

## 4. Conclusions

1. The presence of high contents of asphaltene in heavy crude oil is a critical factor that impacts the dewatering process. At an asphaltene concentration of 0.5 g/L, the dehydration rate is 67%, with fewer droplets and a thinner interface film, resulting in poor stability. As the asphaltene concentration increases, the dehydration rate decreases. And at 11.0 g/L, the rate drops to zero. The droplet size becomes more uniform, and the interface film thickens, significantly improving emulsion stability. The asphaltene concentration also increases emulsion viscosity, along with both the storage modulus (G′) and loss modulus (G″). When the asphaltene concentration was 11 g/L, G′ and G″ reaches 1033 Pa·s and G″ 416 Pa·s, with G′ exceeding G″, suggesting elastic-dominated behavior. The average TSI value of 0.36 further confirms that the emulsion is more stable and very difficult to break;
2. The emulsifying ability of asphaltenes decreases significantly with increased aromaticity in the oil phase. In the toluene–n-heptane emulsion system, the critical aggregation concentrations (CACs) of asphaltenes are 0.05 g/L, 0.8 g/L, and 1.2 g/L for emulsions containing 30%, 40%, and 50% toluene, respectively, which indicates that the critical aggregation concentration (CAC) of asphaltenes increases with the toluene concentrations. The enhanced aromaticity of the emulsion increases asphaltene solubility, reducing its adsorption at the oil–water interface, which decreases emulsion stability and promotes emulsion breakdown and dehydration. Therefore, for crude oils with high asphaltene content prone to emulsification, blending with aromatic-rich oils can optimize the emulsion-breaking and dehydration processes. This work provides theoretical and practical guidance for breaking emulsions during crude oil dewatering pretreatment.

## Figures and Tables

**Table 1:** Components of the Napo oriente crude oil used for asphaltenes extraction.

Parameters	Value	Method
Density (20 °C)/g cm^−3^	0.93	ASTM D1217 [22]
Viscosity (50 °C)/mm^2^ s^−1^	59.00	ASTM D445 [23]
Salt/mg Cl^−1^ kg^−1^	101.56	ASTM D6470 [24]
Sulfur content/wt%	0.88	ASTM D5453 [25]
Resins/wt%	24.90	ASTM D4124 [26]
Asphlatenes/wt%	11.40	ASTM D4124 [26]
Saturates/wt%	29.32	ASTM D4124 [26]
Aromatics/wt%	34.38	ASTM D4124 [26]

**Table 2:** Elemental analysis of the asphaltene.

Asphaltene sample	Composition (wt%)				
	C	H	N	S	O (by difference)
	83.31	7.21	1.55	2.35	2.32

## Data Availability

The original contributions presented in this study are included in the article. Further inquiries can be directed to the corresponding authors.
